# Comparison of the Anesthetic Techniques

**DOI:** 10.1155/2015/650684

**Published:** 2015-03-17

**Authors:** Ahmet Eroglu, Alparslan Apan, Engin Erturk, Izhar Ben-Shlomo

**Affiliations:** ^1^Department of Anesthesiology and Intensive Care Medicine, Karadeniz Technical University Faculty of Medicine, 61000 Trabzon, Turkey; ^2^Department of Anesthesiology and Intensive Care Medicine, Giresun University Faculty of Medicine, 28200 Giresun, Turkey; ^3^Zefat Academic College, Baruch Padeh Medical Center, Israel

A great number of anesthetic techniques (general, regional, spinal, epidural, caudal, hypotensive, total intravenous, regional intravenous, inhalation, and nerve blocks) can be used for multiple surgical procedures [1–3]. The effect of anesthetic technique on perioperative outcomes is controversial.

Central neuraxial blocks including spinal, epidural, and caudal anesthesia are regional anesthesia techniques. Regional anesthesia techniques provide important advantages compared with general anesthesia in some surgical procedures [1, 3]. Regional anesthesia is not only performed for adequate anesthesia in the surgical procedures. There are other advantages for the use of regional anesthesia techniques including excellent pain control, reduced side effects, decreased blood loss, improved cardiac and pulmonary function, and shortened stay in the postanesthesia care unit [1–4]. Low doses of spinal anesthesia and intra-articularly administered analgesic provided a better pain relief, a shorter discharge time, and a higher satisfaction for outpatient arthroscopic knee surgery [2]. Epidural technique as a regional anesthesia is one of the important methods for multimodal postoperative pain control [1, 3]. Hypotensive epidural anesthesia is another technique that decreased blood loss in hip surgeries [1]. Caudal anesthesia is commonly performed in pediatric patients for surgical anesthesia and postoperative analgesia. Regional anesthesia would provide excellent pain control and improve outcomes such as decrease in side effects, improvement of pulmonary function, prevention of chronic pain, or reduction in hospital stay. Thus the regional anesthetic techniques and outcome using regional anesthesia for postoperative pain have becoming one of the important fields [4, 5].

Total intravenous anesthesia (TIVA) has been used in some surgeries and it has been compared with other anesthesia techniques. TIVA with propofol can make a positive contribution to preventing ischemia-reperfusion-associated increases in MDA and IMA in tourniquet-related ischemia reperfusion in arthroscopic knee surgery. In scoliosis surgery, the use of TIVA with propofol and remifentanil is associated with decreased neuroendocrine stress responses in the perioperative period when compared with inhalation anesthesia.

Regional intravenous anesthesia (RIVA) is generally preferred for patients who will have upper extremity surgery due to advantages such as providing a blood-free surgery site, rapid onset and termination of the anesthetic effect, lack of necessity of severe sedation, and general anesthesia and easy application. In addition some analgesic drugs to local anesthetics in intravenous regional anesthesia (IVRA) have been published. The addition of 3 mg/kg paracetamol and 50 mg dexketoprofen to lidocaine as adjuvant in RIVA applied for hand and/or forearm surgery created a significant difference clinically.

Nerve blocks are used for postoperative analgesia. Interscalene brachial plexus block (ISB) is used to provide both anesthesia and analgesia for shoulder surgery. In a study the authors showed that the same volume and concentration of bupivacaine and ropivacaine (30 mL of 0.5%) for interscalene brachial plexus block anesthesia produced similar surgical block. When continuing the block with a patient-controlled interscalene analgesia infusion, 0.15% bupivacaine and ropivacaine provided adequate pain relief, similar side effects, and high patient satisfaction after shoulder surgery.

This special issue contains five clinical studies and a review article related to comparison of anesthetic techniques. In the review article the benefits and risks of hypotensive anesthesia during major maxillofacial surgery were compared to those of normotensive anesthesia. The authors reported that controlled hypotension during anesthesia or hypotensive anesthesia is often used in major maxillofacial operations. Reduced blood pressure is advantageous in some settings because it can contribute to a reduction in overall blood loss and improve the surgical field conditions. Since hypotensive anesthesia carries the risk of hypoperfusion to important organs and tissues, mainly the brain, heart, and kidneys, it cannot be applied safely in all patients.

In a clinical study, spinal and general anesthesia were compared for the impact of the surgical environment, especially the sounds of saw and hammer in the operating room, on patient's mood and anxiety after the operation in total knee arthroplasty (TKA). It was reported that sounds of hammer and saw had no evident negative effect on patient's mood and, in operations performed with spinal anesthesia, the patients were found to be more satisfied so that, with known advantages, regional anesthesia was advisable for TKA patients and appropriate sedation can be administered during the operation if needed.

Two clinical studies are related to the comparison of supraglottic airway devices. The aim of a clinical study was to compare the performance of recently released size 1 I-gel with size 1 ProSeal LMA, which is proven to be superior to the classical LMA for small infants and neonates. The study demonstrated that the size 1 I-gel provided an effective and satisfactory airway as the size 1 ProSeal LMA. It may be a good alternative supraglottic airway device for use in small infants and neonates. However, further studies are needed to determine whether it is reliable for aspiration because of the absence of a gastric drainage tube in this size. Another study compared ProSeal, Supreme, and I-gel supraglottic airway devices in terms of oropharyngeal leak pressures and airway morbidities in gynecological laparoscopic surgeries. It was reported that ProSeal, Supreme, and I-gel provided a safe airway in paralyzed and pressure-controlled ventilation administered gynecological laparoscopic surgeries. While initial oropharyngeal leak pressures obtained by I-gel were lower than ProSeal and Supreme, increased oropharyngeal leak pressures over time, ease of placement, and lower airway morbidity were favorable for I-gel.

The local anesthetics used in day-case spinal anesthesia should provide short recovery times. In a clinical study hyperbaric prilocaine and bupivacaine were compared in terms of sensory block resolution and time to home readiness in day-case spinal anesthesia. In the study it was reported that day-case spinal anesthesia with prilocaine 30 mg + 20 *μ*g fentanyl provided faster sensory block resolution and home readiness compared to 7.5 mg bupivacaine + 20 *μ*g fentanyl and the surgical conditions were comparable for perianal surgery.

Dislocation of epidural catheters (EC) may cause early termination of postoperative regional analgesia. In a clinical study the hypothesis that maximum effort in fixation by catheter tunneling and suture decreases the incidence of its dislocation was tested. It was reported that thorough tunneling and suture of thoracic epidural catheters significantly reduced incidence and extent of catheter dislocation and potentially that of bacterial contamination.

The outcomes of the comparison of anesthetic techniques are multifarious. In the future more researches are needed to explain the potential mechanisms for these outcomes.

*Ahmet Eroglu* *Ahmet Eroglu* *Alparslan Apan* *Alparslan Apan* *Engin Erturk* *Engin Erturk* *Izhar Ben-Shlomo* *Izhar Ben-Shlomo*

## References

[1] Mauermann W. J., Shilling A. M., Zuo Z. (2006). A comparison of neuraxial block versus general anesthesia for elective total hip replacement: a meta-analysis. *Anesthesia & Analgesia*.

[2] Eroglu A., Saracoglu S., Erturk E., Kosucu M., Kerimoglu S. (2010). A comparison of intraarticular morphine and bupivacaine for pain control and outpatient status after an arthroscopic knee surgery under a low dose of spinal anaesthesia. *Knee Surgery, Sports Traumatology, Arthroscopy*.

[3] Macfarlane A. J. R., Prasad G. A., Chan V. W. S., Brull R. (2009). Does regional anaesthesia improve outcome after total hip arthroplasty? A systematic review. *British Journal of Anaesthesia*.

[4] Casati A., Borghi B., Fanelli G. (2003). Interscalene brachial plexus anesthesia and analgesia for open shoulder surgery: a randomized, double-blinded comparison between levobupivacaine and ropivacaine. *Anesthesia and Analgesia*.

[5] Memtsoudis S. G., Sun X., Chiu Y. L. (2013). Perioperative comparative effectiveness of anesthetic technique in orthopedic patients. *Anesthesiology*.

